# Pan-cancer analysis of tumor metabolic landscape associated with genomic alterations

**DOI:** 10.1186/s12943-018-0895-9

**Published:** 2018-10-17

**Authors:** Hongyoon Choi, Kwon Joong Na

**Affiliations:** 1 Department of Nuclear Medicine, Seoul National University Hospital, 101 Daehak-ro, Jongno-gu, Seoul, 03080 Republic of Korea; 2 Department of Thoracic and Cardiovascular Surgery, Seoul National University Hospital, 101 Daehak-ro, Jongno-gu, Seoul, 03080 Republic of Korea

**Keywords:** Tumor mutation burden, Metabolic landscape, Cancer metabolism, Pan-cancer analysis, Driver gene mutation

## Abstract

Although metabolic alterations are one of the hallmarks of cancer, there is a lack of understanding of how metabolic landscape is reconstituted according to cancer progression and which genetic alterations underlie its heterogeneity within cancer cells. Here, the configuration of the metabolic landscape according to genetic alteration is examined across 7648 subjects representing 29 cancers. The metabolic landscape and its reconfiguration according to the accumulated mutation maintained characteristics of their tissue of origin. However, there were some common patterns across cancers in terms of the association with cancer progression. Carbohydrate and pyrimidine metabolism showed the highest positive correlation with tumor metabolic burden and they were also common poor prognostic pathways in several cancer types. We additionally examined whether genetic alterations associated with the heterogeneity of metabolic landscape. Genetic alterations associated with each metabolic pathway differed between cancers, however, they were a part of cancer drivers in most cancer types.

**Electronic supplementary material:**

The online version of this article (10.1186/s12943-018-0895-9) contains supplementary material, which is available to authorized users.

Cancer cells reorganize their metabolism to fulfill their biosynthetic requirements for tumor growth and proliferation [1]. Besides activation of aerobic glycolysis, other metabolic pathways including nucleotide, amino acid, and lipid metabolism are also activated in cancer cells to produce biosynthetic building blocks for malignant cellular proliferation although the degree of metabolic activation is diverse [2, 3]. Although recent studies have elucidated the metabolic reprogramming of cancer cells compared with normal tissues and its biological implications [2, 4], the configuration of metabolic landscape according to cancer progression in terms of tumor mutational burden (TMB) and clinical outcome is yet unclear. Moreover, the diversity of cancer cell metabolism is hardly explained by the conventional analogy of genetic alteration model that tumor metabolism simply supports tumor growth and proliferation [5, 6]. A comprehensive understanding of genetic alterations which underlie the heterogeneity of the metabolic landscape of cancer cells can elucidate therapeutic targets in terms of cancer metabolism.

Here, we aim to investigate the metabolic landscape of multiple cancer types and its configuration according to the progression. We also aim to find answers for whether there are common features in genetic alterations related to the metabolic landscape across cancer types. An integrated analysis of genomic and transcriptomic profiles of 29 different solid cancers was performed. We demonstrate that some common metabolic pathways exhibit the close relationship with TMB and clinical outcome across several cancer types. We also show that genetic alterations associated with cancer metabolic heterogeneity were a part of cancer drivers in most cancer types.

## Results and Discussions

### Metabolic landscape specific for cancer types

We compared the median values of metabolic signatures manually curated considering cancer-related pathways for each cancer type (Additional file 1: Figure S1A). Metabolic profiles of all cancers were visualized by t-SNE [7] (Additional file 1: Figure S1B). Metabolic profiles of cancer tissues were clustered according to each cancer type. Additionally, metabolic profiles were closely mapped with those of same histologic types (e.g. HNSC, LUSC, and CESC clusters.) Of note, metabolic profiles of TCGT and CHOL showed heterogeneous metabolic characteristics. TGCT showed two large clusters, which correspond to histologic subtypes, and CHOL showed more heterogeneous metabolic profiles than others (Additional file 1: Figure S2).

### Metabolic reconstitution according to mutational burden

**Fig. 1:**
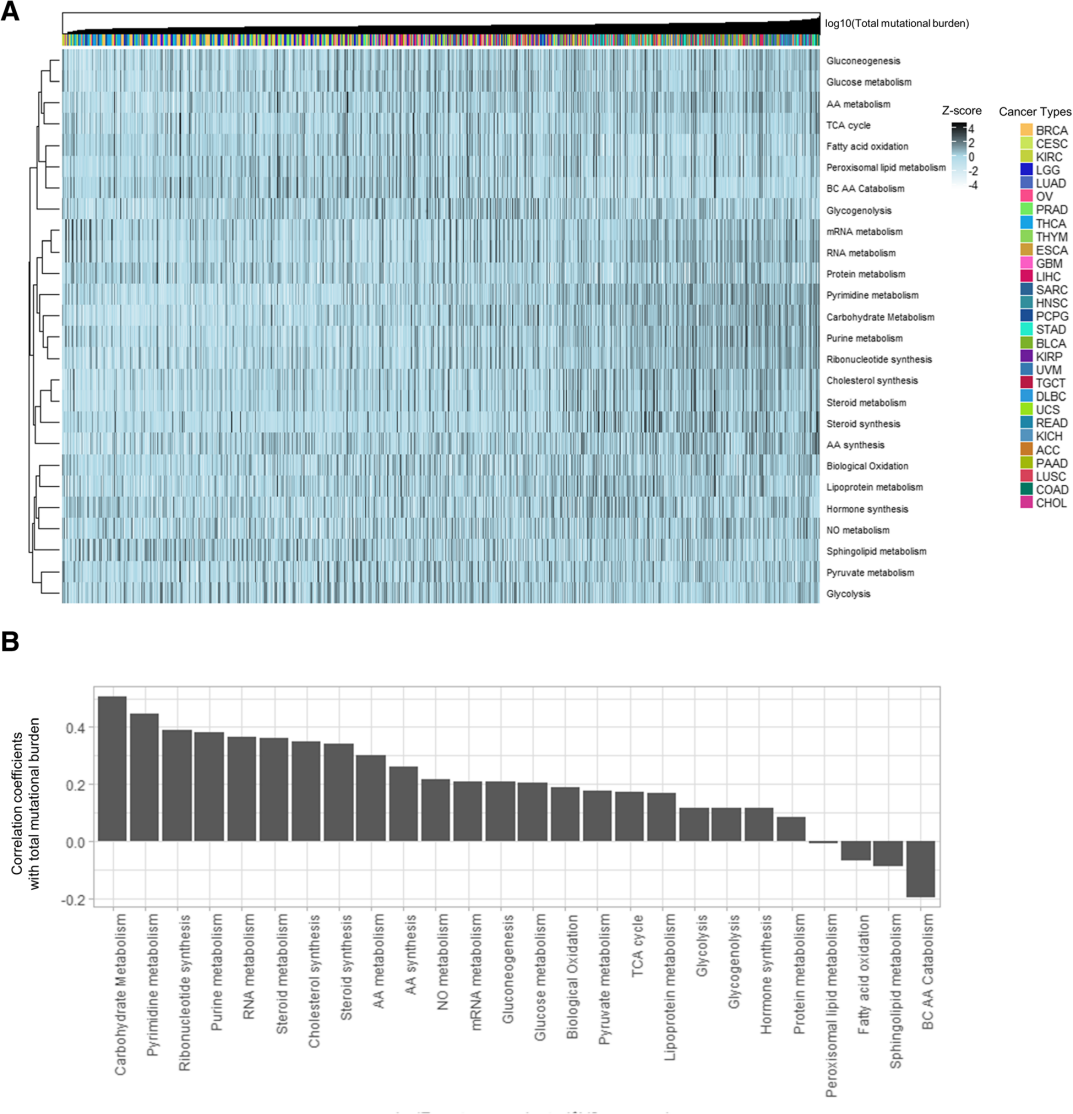
Pan-cancer association of tumor mutation burden and metabolic landscape. **a** The enrichment scores of metabolic pathways are depicted according to total mutation burden for all samples. Cancer type is shown as different color in the barplot above the heatmap. **b** The correlation coefficient for total mutation burden and each metabolic pathway is presented. Carbohydrate and pyrimidine metabolism show high positive correlation and most of pathways related to lipid metabolism and oxidative process show negative correlation with tumor mutation burden

Metabolic signatures of all samples were ordered by TMB and presented by a heatmap (Fig. 1a). Carbohydrate metabolism showed the highest correlation with TMB followed by pyrimidine metabolism (Fig. 1b; *p* < 0.05 for all pathways). Of note, glycolysis, the well-known cancer metabolic feature, showed relatively weak correlation with TMB. LUSC showed the highest carbohydrate metabolism and TMB among 29 cancer types (Additional file 1: Figure S3). We could find a trend of increased carbohydrate and pyrimidine metabolism according to increased TMB across cancer types (Additional file 1: Figure S4). Of note, carbohydrate metabolism pathways included broad metabolic pathways related to energy metabolism including glycogen, glucose, disaccharides, pentose phosphate, and glycosaminoglycan, thus, the association with TMB suggested comprehensive changes in various energy metabolism pathways. We also investigated whether cancer types differ in the association between the metabolic landscape and TMB. Most cancer types showed a positive correlation between TMB and carbohydrate metabolism, while THYM and UVM showed a negative correlation (Additional file 1: Figure S5). Notably, TMB of these two cancer types was relatively lower than other cancer types.

Recently, TMB has been regarded as an important predictive biomarker for cancer immunotherapy, based on the idea that highly mutated tumors harboring more neoantigens can be targets of activated immune cells [8, 9]. In this regard, metabolic reconfiguration according to TMB can be an additional biomarker for cancer immunotherapy as tumor metabolism can be noninvasively and macroscopically estimated by PET. Moreover, the important metabolic signatures of each cancer type could help us find appropriate surrogate markers for PET radiotracers.

### Metabolic landscape reveals prognostic pathways

**Fig. 2:**
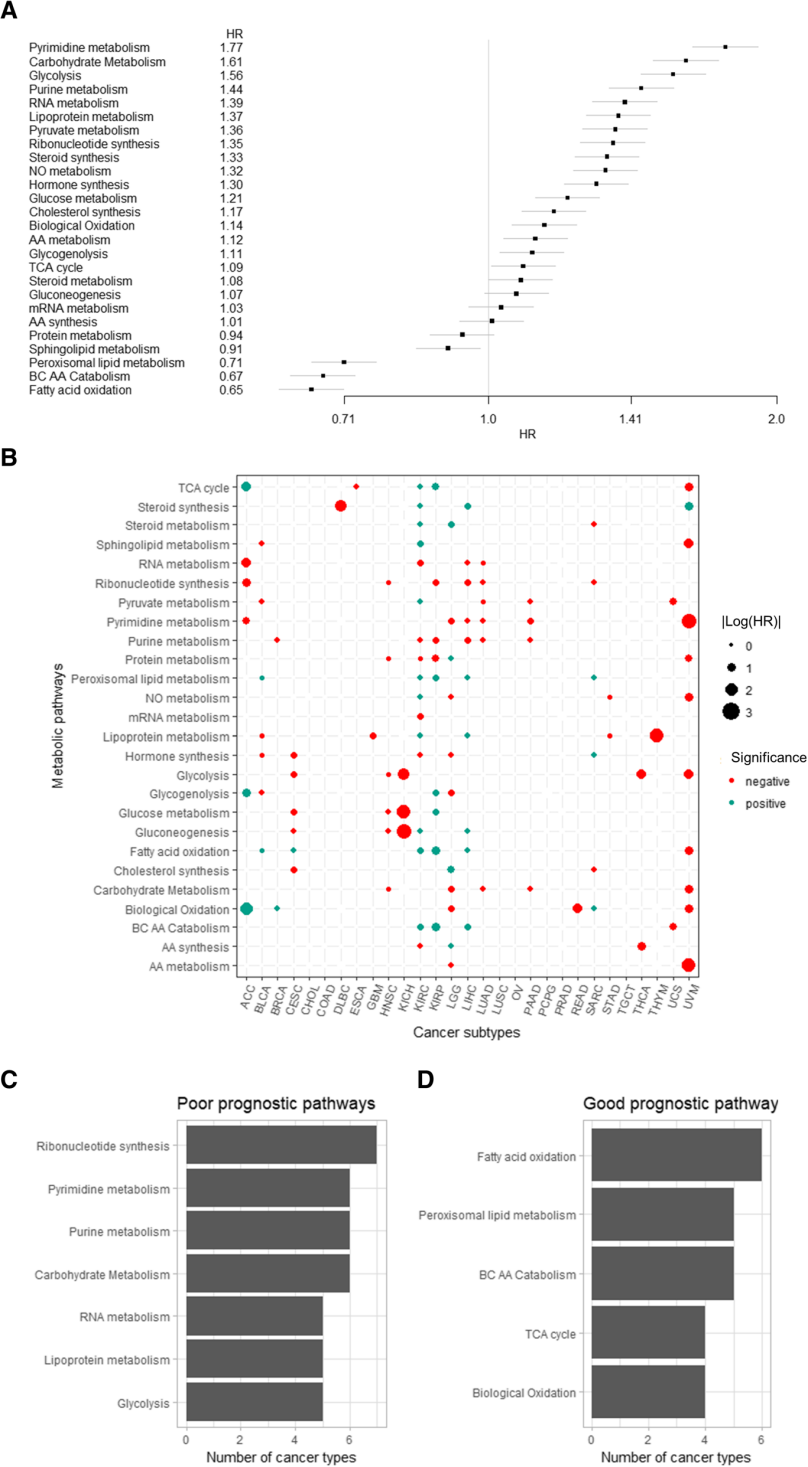
Prognostic significance of metabolic pathways. **a** Hazard ratios from pan-cancer analysis of each metabolic pathways to overall survival are shown. **b** The bubble plot shows the result of Cox proportional analysis for each cancer type. Only significant (*p* < 0.05) metabolic pathways are shown in this plot. The size of circle represents the log-scaled hazard ratio, and the color of circle represents negative (red) or positive (blue) prognostic significance. **c, d** Frequency of metabolic pathways found negative (**c**) or positive (**d**) prognostic significance

Several metabolic signatures were associated with overall survival across all cancer samples (Fig. 2a; *p* < 0.05 for all pathways). In general, carbohydrate and nucleotide metabolism were associated with poor prognosis. Specifically, poor prognostic metabolic signatures include carbohydrate metabolism, ribonucleotide synthesis, pyrimidine metabolism, purine metabolism, and glycolysis, whereas good prognostic signatures include peroxisomal lipid metabolism, fatty acid oxidation, and biological oxidation.

Metabolic signatures significantly associated with prognosis for each cancer type were presented in Fig. 2b. The most common metabolic signatures significantly associated with poor prognosis were ribonucleotide synthesis (7 of 29 cancers), followed by pyrimidine, purine, and carbohydrate metabolism (6 or 29 cancers) (Fig. 2c). The metabolic signatures significantly associated with good prognosis were fatty acid oxidation (6 of 29 cancers), peroxisomal lipid metabolism, and branched chain amino acids catabolism (5 of 29 cancers) (Fig. 2d).

In brief, the increased metabolic activity of carbohydrate and nucleotides in cancer was associated with poor prognosis as well as the progression of cancer in terms of TMB as found in previous results. Furthermore, our results were similar with the previous report, which showed that glycolysis, ribonucleotide, pyrimidine, and purine metabolism were associated with poor prognosis, and mitochondrial fatty acid oxidation and peroxisomal lipid metabolism were associated with good prognosis [2]. These results support the idea that the link between biological stepwise mutational progression, metabolic landscape reconfiguration, and clinical progression of cancer. Accordingly, aggressive malignant phenotypes of cancer cells obtained by accumulated mutations change metabolic phenotypes to demand energy source represented by carbohydrate and elements for proliferation represented by nucleotide.

### Metabolism-related genetic alterations are mostly cancer drivers

**Fig. 3:**
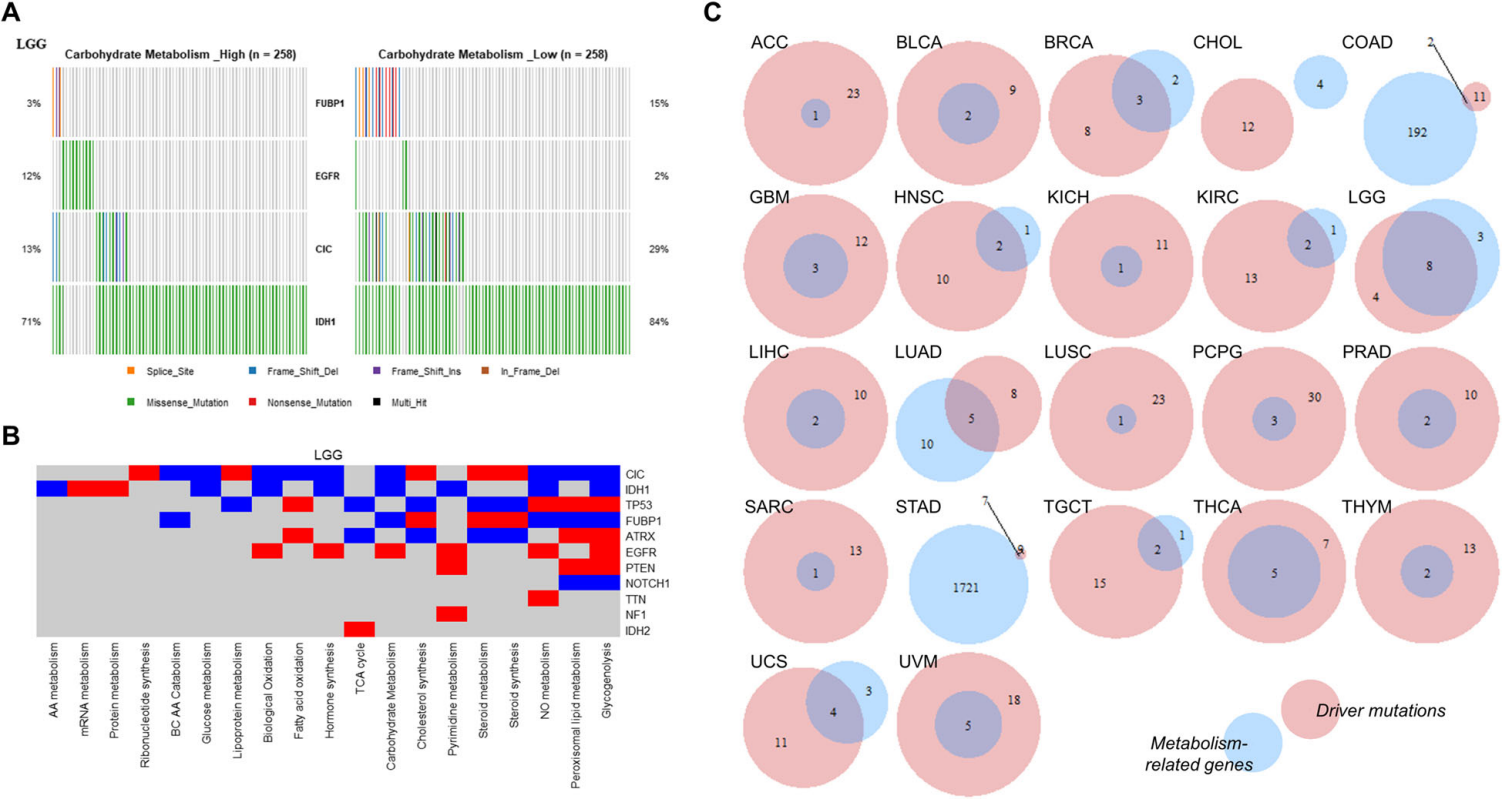
Metabolic-related genes and driver gene mutation. **a** Carbohydrate metabolism-related genes in LGG. Each oncoplot shows the genomic alteration of each group from LGG divided by the median enrichment scores of carbohydrate metabolism. Four genes (FUBP1, CIC, IDH1, and EGFR) were identified as differentially mutated genes between two groups. **b** All metabolic-related genes in LGG. Red color represents high mutation burden in high metabolic signature, and blue color represents low mutation burden in high metabolic signature. **c** Venn diagram showing the number of metabolic-related genes and driver gene mutation for each cancer subtype

As cancer cells exhibit various metabolic landscapes even in same cancer types, genetic alterations could underlie this variety. We tried to examine the genetic alterations related to the metabolic landscape of each cancer type to investigate whether there is a common feature in the metabolism-related genetic alterations across several cancer types. An example of carbohydrate metabolism-related genetic alterations of LGG is presented (Fig. 3a.) The tumors with high carbohydrate metabolism showed significantly more mutations of EGFR and fewer mutations of FUBP1, CIC, and IDH1. The genetic alterations of LGG related to other metabolic signatures are summarized in Fig. 3b. We defined differentially-mutated genes of tumors for at least one metabolic signature as the metabolism-related genes. All metabolism-related genes of each cancer type are presented in Additional file 1: Figure S6. Seven cancer types (CESC, DLBC, ESCA, KIRP, OV, PAAD, and READ) showed no metabolism-related gene.

We further examined whether the metabolism-related genes were cancer driver genes. All metabolism-related genes of 12 cancer types (ACC, BLCA, GBM, KICH, LIHC, LUSC, PCPG, PRAD, SARC, THCA, THYM, and UVM) were included in cancer drivers (Fig. 3c). In other cancer types, most of the metabolism-related genes overlapped with cancer drivers except STAD, COAD, and CHOL. The metabolism-related genes of CHOL did not overlap with cancer drivers. Notably, metabolic profiles of CHOL were highly heterogeneous (Additional file 1: Figure S2) as a previous result. STAD and COAD showed a large number of the metabolism-related genes than other cancer types. Since they are known to include tumors with MSI which is associated with hypermutation [10, 11], we examined the relationship between metabolic profiles of the two cancer types and MSI status. As shown in Additional file 1: Figure S7, tumors with high metabolic signatures including pyrimidine metabolism and glucose metabolism were clustered in those with MSI.

According to the results, cancer transcripts and protein networks consisting of metabolic pathways may be changed by cancer drivers instead of alterations of genes participated in the networks. It corresponds to the Darwinian selection of cancer cells which carry driver mutations facilitate cellular survival and growth by changing a favorable metabolic landscape [12]. The results also suggest that the metabolic change as a hallmark of cancer may be one of the tumorigenesis processes caused by genetic alterations rather than an independent process.

## Conclusions

Among several metabolic signatures, carbohydrate metabolism representing overall nutrient demand and nucleotide metabolism showed the closest association with TMB and clinical outcome. Genetic alterations closely associated with metabolic heterogeneity were a part of cancer drivers in most cancers rather than genes affiliated to metabolic pathways. It supports the role of cancer drivers in the evolution process in constituting metabolic landscape appropriate for tumor growth. Our database of the metabolic landscape (https://choih.shinyapps.io/metabolicsignatures) according to cancer type and its reconstitution according to cancer progression can contribute to developing appropriate diagnostics and therapeutics targeting metabolism for cancer subtypes.

## Additional file

Additional file 1: Materials and methods, supplementary figures and supplementary tables. (PDF 1650 kb)

## Abbreviations

MSI: Microsatellite instability
TCGA: The Cancer Genome Atlas
TMB: Tumor mutation burden
t-SNE: T-Distributed Stochastic Neighbor Embedding

## Abbreviations for cancer type

ACC: Adrenocortical carcinoma
BLCA: Bladder urothelial carcinoma
CESC: Cervical and endocervical cancers
CHOL: Cholangiocarcinoma
COAD: Colon adenocarcinoma
DLBC: Diffuse Large B-cell Lymphoma
ESCA: Esophageal carcinoma
GBM: Glioblastoma multiforme
HNSC: Head and Neck Squamous Cell Carcinoma
KICH: Kidney Chromophobe
KIRP: Kidney renal papillary cell carcinoma
LGG: Brain low grade glioma
LIHC: Liver hepatocellular carcinoma
LUSC: Lung Squamous Cell Carcinoma
OV: Ovarian serous cystadenocarcinoma
PAAD: Pancreatic adenocarcinoma
PCPG: Pheochromocytoma and Paraganglioma
PRAD: Prostate adenocarcinoma
READ: Rectum adenocarcinoma
SARC: Sarcoma
STAD: Stomach adenocarcinoma
TCGT: Testicular Germ Cell Tumors
THCA: Thyroid carcinoma
THYM: Thymoma
UVM: Uveal Melanoma

## Abbreviations for genes

CIC: Capicua Transcriptional Repressor
EGFR: Epidermal growth factor receptor
FUBP1: Far Upstream Element Binding Protein 1
IDH1: Isocitrate dehydrogenase 1
