# Supplementary material for: Pan-cancer analysis of tumor metabolic landscape associated with genomic alterations
Source: Mol Cancer. 2018 Oct 17;17:150. doi: 10.1186/s12943-018-0895-9 (PMC6192220; doi:10.1186/s12943-018-0895-9)
Supplement: Supplementary file 1 — Materials and methods, supplementary figures and supplementary tables. (PDF 1650 kb) [file 12943_2018_895_MOESM1_ESM.pdf]

SUPPLEMENTARY MATERIALS

Pan-cancer analysis of tumor metabolic landscape associated with genomic alterations

Materials and Methods

Supplementary Figures

Supplementary Tables

Supplementary References

Materials and Methods

Preprocessing for transcriptomic and genomic data of TCGA

We used publicly available cancer genome and transcriptome data from the TCGA projects. Using 'TCGABiolinks' R package [1], we downloaded the level three RNA sequence data of 32 solid cancers from TCGA data portal (https://portal.gdc.cancer.gov/) on Dec 12th, 2017, obtained with Illumina HiSeq RNASeqV2 (Illumina, San Diego, CA, USA). For each type of TCGA projects, we normalized mRNA transcripts using 'TCGAAnalyze_Normalization' function and filtered low expression genes with 'TCGAanalyze_Filtering' function. We merged transcriptome data of each TCGA projects into one large-scale expression matrix for pan-cancer analysis. Clinical information, including vital status, follow-up time, and time of death was also collected in the same manner. The information for microsatellite instability (MSI) or hypermutated status of both STAD and COAD were downloaded from the website of The cBioPortal for Cancer Genomics (http://www.cbioportal.org) on Dec 23th, 2017. We downloaded pre-compiled, curated somatic mutations data of 32 solid cancer types from TCGA projects, provided by 'TCGAmutations', as a R data package (https://github.com/PoisonAlien/TCGAmutations). The pre-compiled data were derived from the latest analysis data (January 28th, 2016) which was downloaded via Broad Institute GDAC Firehose pipeline. Mutation data were analyzed and summarized using maftools package [2]. We excluded three cancer subtypes (malignant mesothelioma, uterine corpus endometrial carcinoma, and skin cutaneous melanoma) for the analysis due to insufficient mutation data in the pre-compiled data; conclusively, genomic and transcriptomic data from total 29 cancer subtypes were used for the analysis in the present study.

Calculating enrichment scores of metabolic pathways

To analyze cancer type-specific metabolic landscape, we used 26 metabolic pathways defined by Reactome [3] across 29 cancer types. Single sample gene set enrichment analysis was then applied against the curated gene sets of the Reactome metabolic pathways to define metabolic profiles of each cancer samples. We implemented single sample gene set enrichment analysis [4] using the curated gene sets from canonical pathways (MSigDB C2, Broad Institute; version 3.0) with GSVA R/Bioconductor package [5, 6]. To identify the functional enrichment scores of metabolic pathways of each sample, we extracted the enrichment scores of 26 Reactome pathways which were related to metabolism (Supplementary table 1) [3]. The enrichment scores of Reactome metabolic pathways were normalized by z-score across all samples. In total, we analyzed 7648 transcriptomic and genomic data (Number of samples and abbreviation for each cancer type is summarized in Supplementary Table 2).

Two-dimensional metabolic landscape mapping

To visualize differences in metabolic landscape, a dimension reduction method, t-Distributed Stochastic Neighbor Embedding (t-SNE), was used [7]. Briefly, using t-SNE, similar samples are modeled by nearby points to maintain local similarity. The similarity between a sample and other samples is defined by Gaussian with a number of neighbors, perplexity. We set the perplexity to 30.

Differentially mutated genes according to each metabolic pathway

For each metabolic signatures, the samples of each tumor type were divided into two groups according to the median value of the enrichment score. The differentially mutated

genes between low and high enrichment scores group were evaluated by a fisher test on all genes. The genes with p-value corrected by false discovery rate under 0.05 were regarded as significantly differentially mutated genes.

Cancer driver mutation

We compared significantly differentially mutated genes for metabolic signatures and cancer drivers. Cancer drivers were identified by a pre-computed drivers using various algorithms deposited by DriverDBv2 (http://driverdb.tms.cmu.edu.tw/driverdbv2/) [8]. This database provides cancer drivers identified by multiple algorithms and we can choose the number of algorithms to find duplicate driver genes. We chose the maximal number of computational algorithms for each cancer type to define cancer drivers.

Statistical analysis

The correlation analysis between the TMB and enrichment scores of metabolic pathways was performed by the Spearman's correlation test. The prognostic property of each enrichment score of metabolic pathways on overall survival was evaluated by using the Cox proportional regression analysis in each cancer subtypes as well as pan-cancer data. All statistical analyses were done within the R program (v3.4.3).

Supplementary Figures

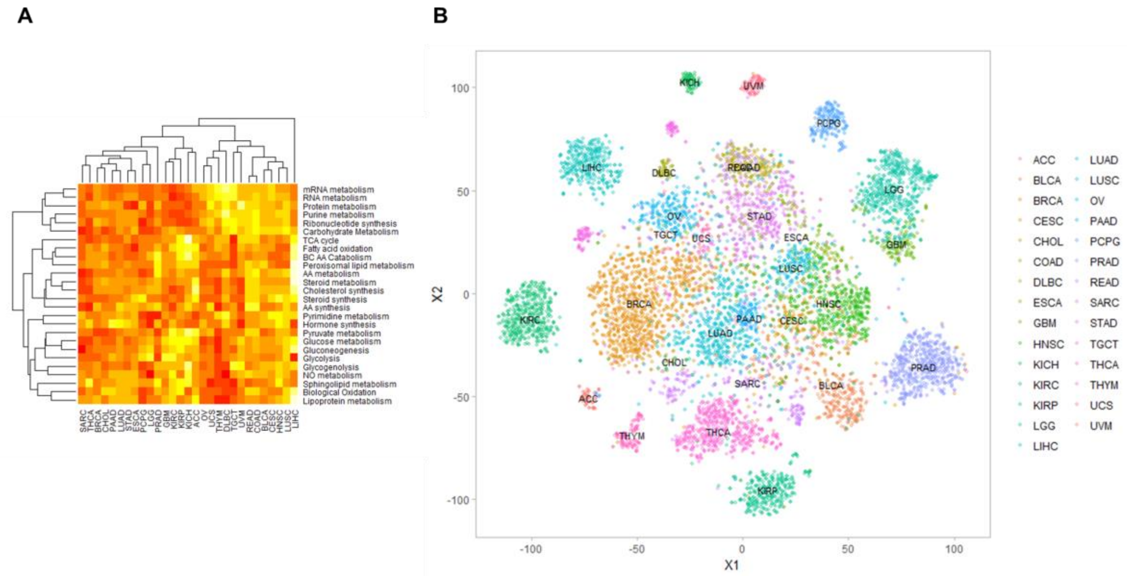

Supplementary Figure 1. Metabolic landscape of 29 types of cancer. (A) The heatmap depicting the median enrichment scores of 26 Reactome metabolic pathways for each cancer type. Red, yellow color represents high, low enrichment scores, respectively. (B) All samples were mapped to the two-dimensional projection using t-Distributed Stochastic Neighborhood Embedding based on the enrichment scores of metabolic pathways. Each cancer type is depicted in different color.

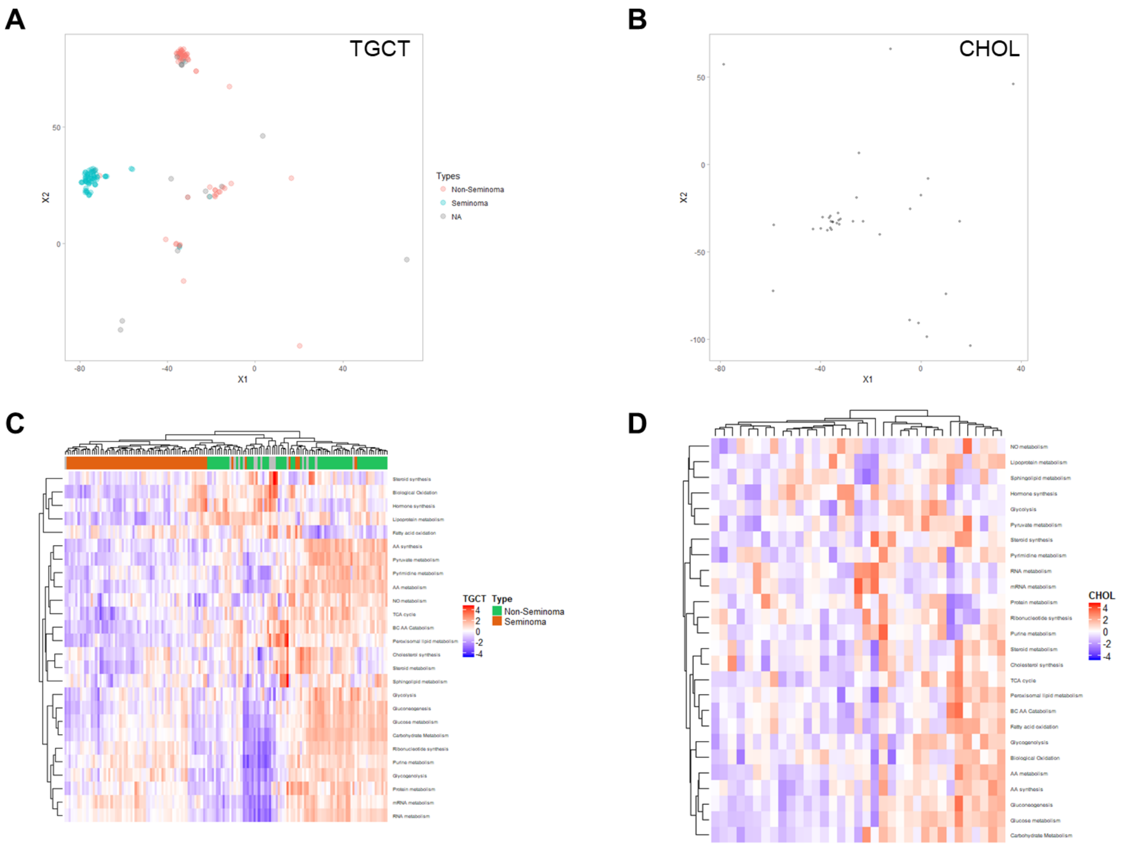

Supplementary Figure 2. Metabolic landscape of testicular germ cell tumor (TCGT) and cholangiocardinoma (CHOL). (A, B) t-Distributed Stochastic Neighbor Embedding analysis of TCGT (A) and CHOL (B) data sets relative to metabolic signatures. (C, D) The heatmap depicting metabolic signatures of TCGT (C) and CHOL (D). The histologic types of testicular germ cell tumor are shown for each sample (above the heatmap).

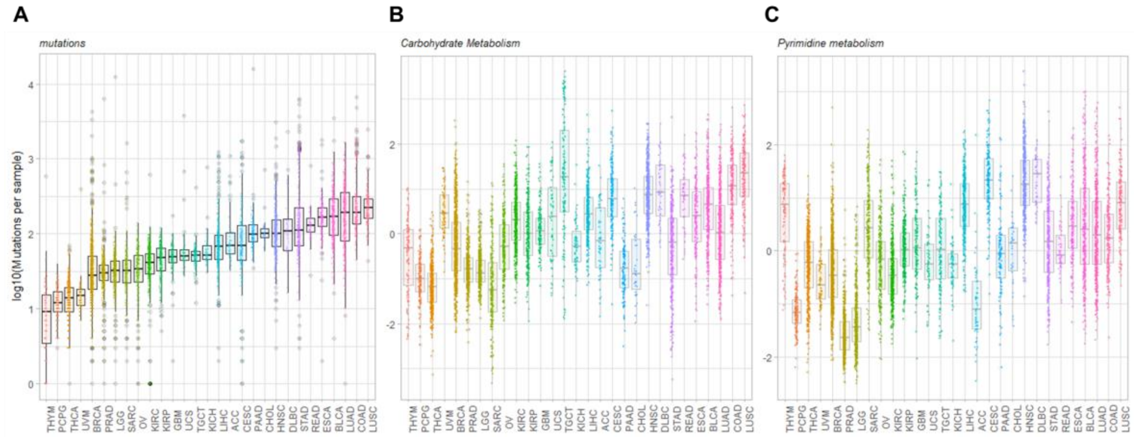

Supplementary Figure 3. Distribution of total mutation burden carbohydrate, and pyrimidine metabolism in each cancer type. Total mutation burden (A), enrichment scores of carbohydrate (B) and pyrimidine metabolism (C) across 29 cancers.

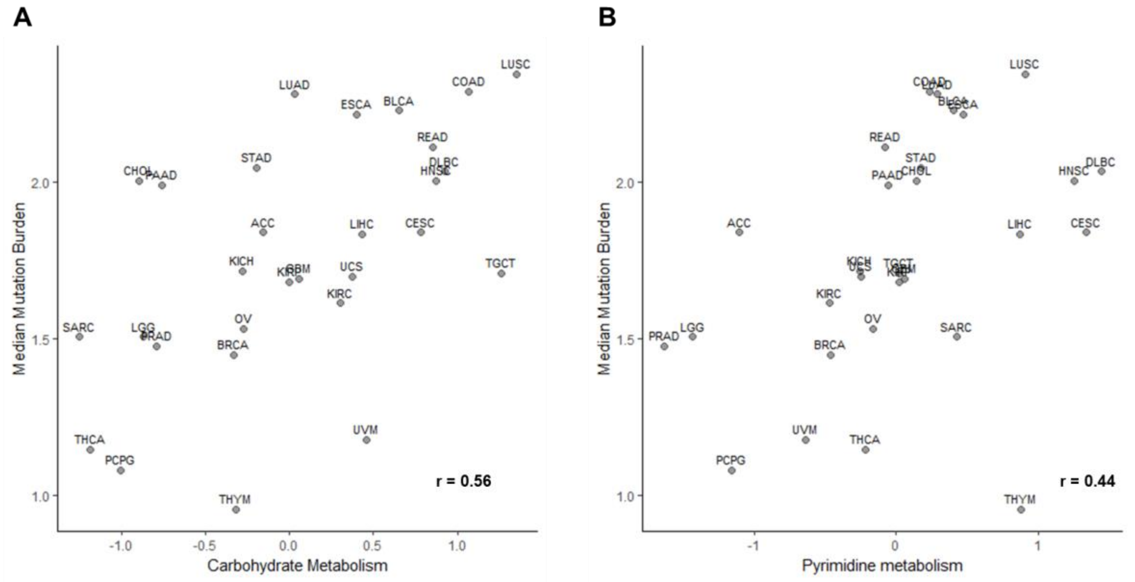

Supplementary Figure 4. Association between total mutation burden and carbohydrate, pyrimidine metabolism. The points of scatter plot representing the median value of total mutation burden of each cancer type (y-axis) and carbohydrate (A), and pyrimidine (B) metabolism (x-axis). The correlation coefficient (r value) for each plot was calculated from pan-cancer analysis.

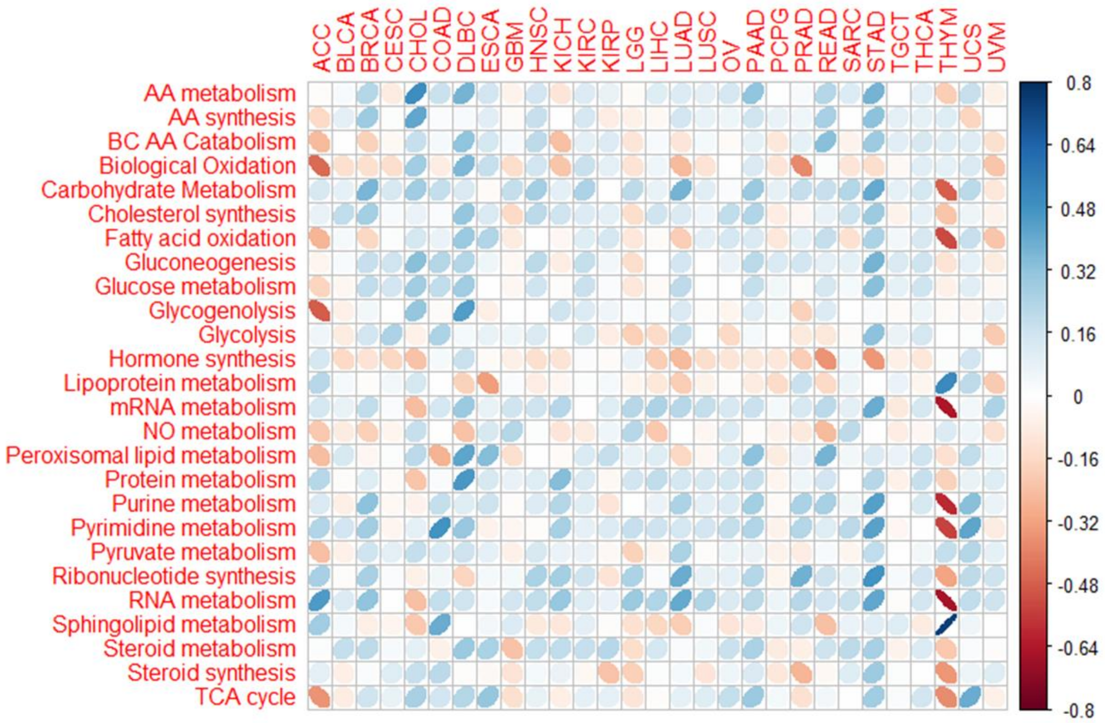

Supplementary Figure 5. Correlation of the enrichment scores of metabolic pathways and tumor mutation burden across 29 cancers. The Spearman's rank correlation matrix representing the relationship between enrichment scores of 26 metabolic pathways from Reactome and tumor mutation burden across 29 cancers. The colors of the scale bar denote the nature of the correlation with positive correlation in blue scale and negative correlation in red scale. The ellipses have their eccentricity parametrically scaled to the correlation coefficient value.

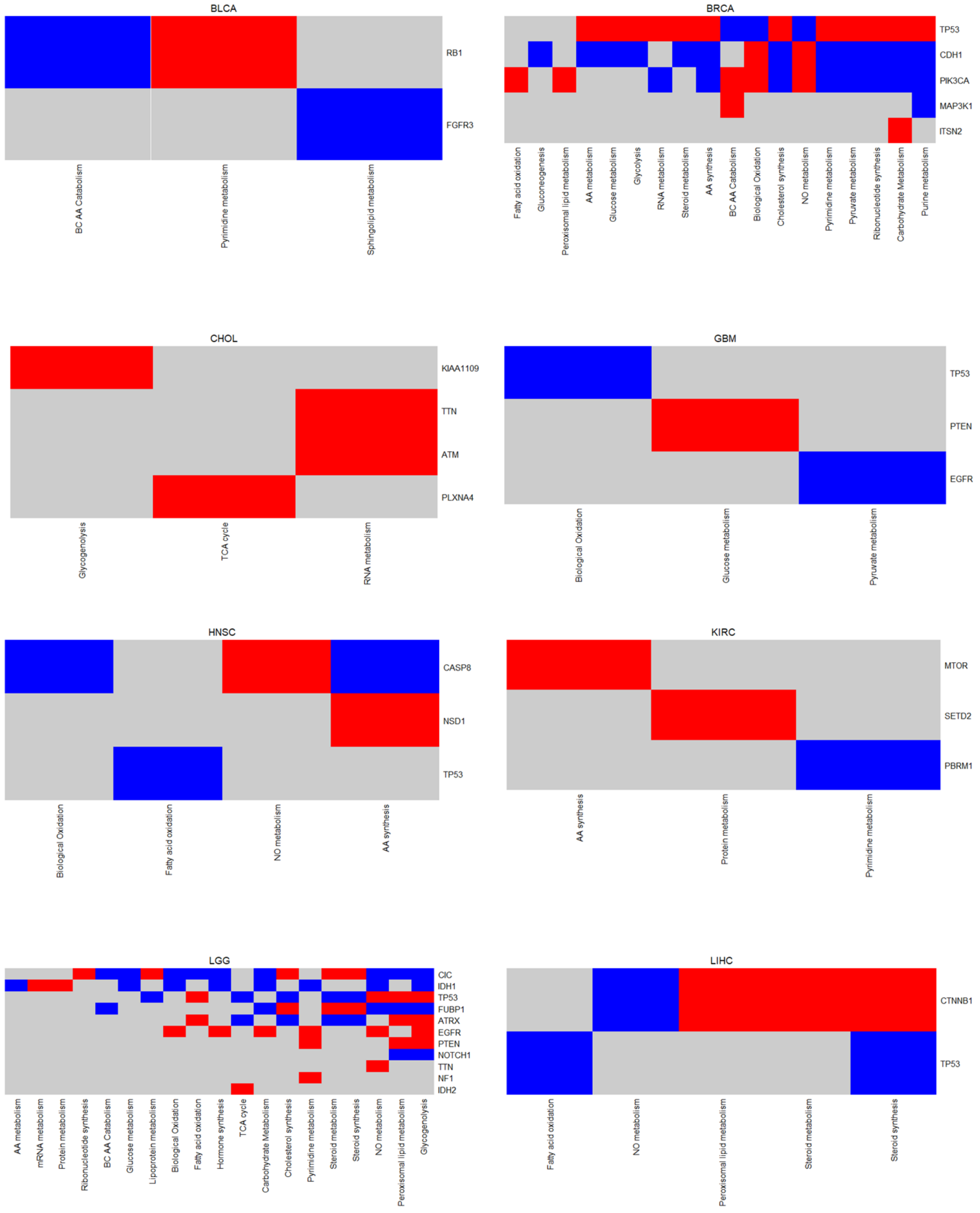
BLCA
RB1
FGFR3
BC AA Catabolism
Pyrimidine metabolism
Sphingolipid metabolism
BRCA
TP53
CDH1
PIK3CA
MAP3K1
ITSN2
Fatty acid oxidation
Gluconeogenesis
Peroxisomal lipid metabolism
AA metabolism
Glucose metabolism
Glycolysis
RNA metabolism
Steroid metabolism
AA synthesis
BC AA Catabolism
Biological Oxidation
Cholesterol synthesis
NO metabolism
Pyrimidine metabolism
Pyruvate metabolism
Ribonucleotide synthesis
Carbohydrate Metabolism
Purine metabolism
CHOL
KIAA1109
TTN
ATM
PLXNA4
Glycogenolysis
TCA cycle
RNA metabolism
GBM
TP53
PTEN
EGFR
Biological Oxidation
Glucose metabolism
Pyruvate metabolism
HNSC
CASP8
NSD1
TP53
Biological Oxidation
Fatty acid oxidation
NO metabolism
AA synthesis
KIRC
MTOR
SETD2
PBRM1
AA synthesis
Protein metabolism
Pyrimidine metabolism
LGG
CIC
IDH1
TP53
FUBP1
ATRX
EGFR
PTEN
NOTCH1
TTN
NF1
IDH2
AA metabolism
mRNA metabolism
Protein metabolism
Ribonucleotide synthesis
BC AA Catabolism
Glucose metabolism
Lipoprotein metabolism
Biological Oxidation
Fatty acid oxidation
Hormone synthesis
TCA cycle
Carbohydrate Metabolism
Cholesterol synthesis
Pyrimidine metabolism
Steroid metabolism
Steroid synthesis
NO metabolism
Peroxisomal lipid metabolism
Glycogenolysis
LIHC
CTNNB1
TP53
Fatty acid oxidation
NO metabolism
Peroxisomal lipid metabolism
Steroid metabolism
Steroid synthesis

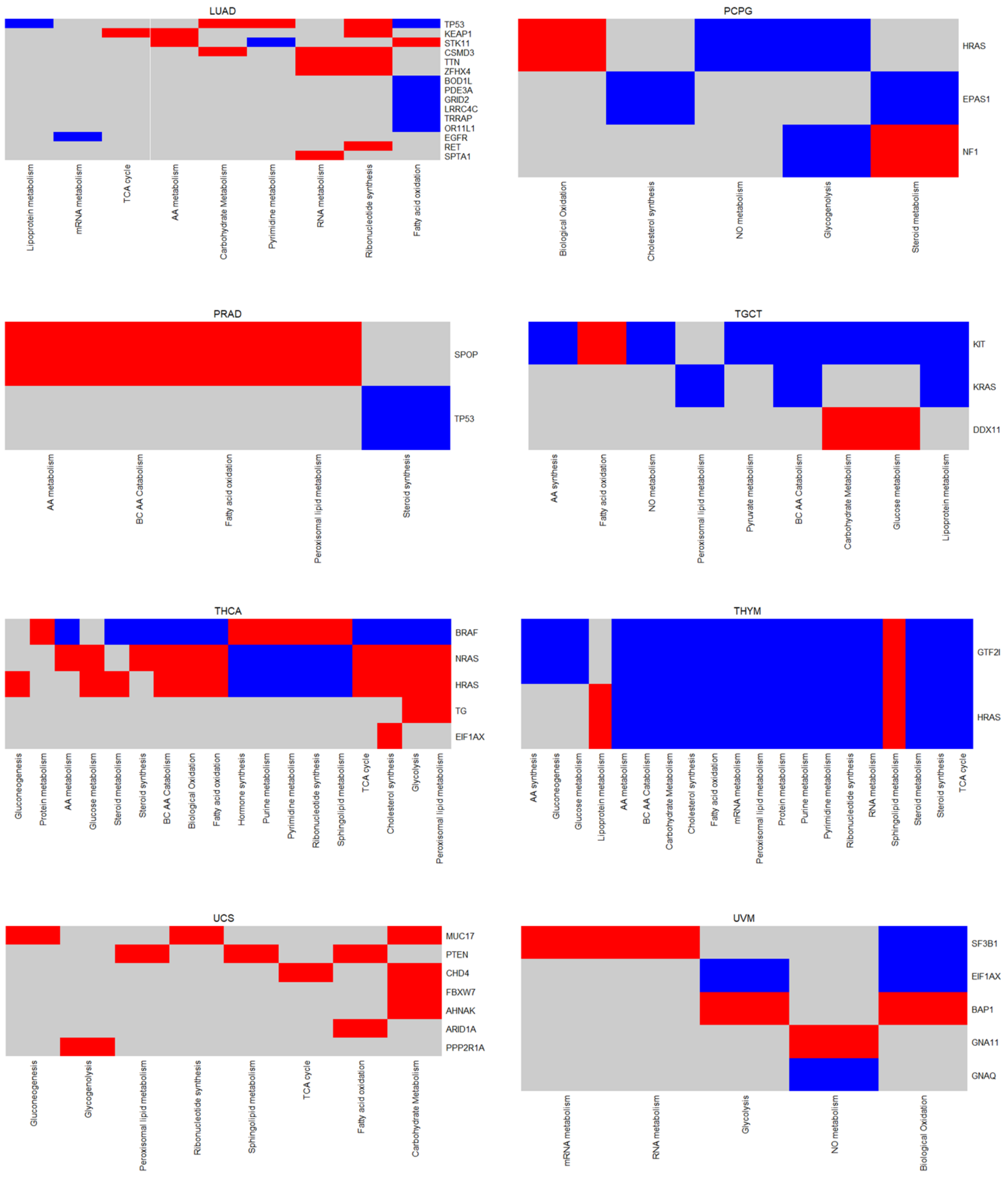
LUAD
TP53
KEAP1
STK11
CSMD3
TTN
ZFHX4
BOD1L
PDE3A
GRID2
LRRC4C
TRRAP
OR11L1
EGFR
RET
SPTA1
Lipoprotein metabolism
mRNA metabolism
TCA cycle
AA metabolism
Carbohydrate Metabolism
Pyrimidine metabolism
RNA metabolism
Ribonucleotide synthesis
Fatty acid oxidation
PCPG
HRAS
EPAS1
NF1
Biological Oxidation
Cholesterol synthesis
NO metabolism
Glycogenolysis
Steroid metabolism
PRAD
SPOP
TP53
AA metabolism
BC AA Catabolism
Fatty acid oxidation
Peroxisomal lipid metabolism
Steroid synthesis
TGCT
KIT
KRAS
DDX11
AA synthesis
Fatty acid oxidation
NO metabolism
Peroxisomal lipid metabolism
Pyruvate metabolism
BC AA Catabolism
Carbohydrate Metabolism
Glucose metabolism
Lipoprotein metabolism
THCA
BRAF
NRAS
HRAS
TG
EIF1AX
Gluconeogenesis
Protein metabolism
AA metabolism
Glucose metabolism
Steroid metabolism
Steroid synthesis
BC AA Catabolism
Biological Oxidation
Fatty acid oxidation
Hormone synthesis
Purine metabolism
Pyrimidine metabolism
Ribonucleotide synthesis
Sphingolipid metabolism
TCA cycle
Cholesterol synthesis
Glycolysis
Peroxisomal lipid metabolism
THYM
GTF2I
HRAS
AA synthesis
Gluconeogenesis
Glucose metabolism
Lipoprotein metabolism
AA metabolism
BC AA Catabolism
Carbohydrate Metabolism
Cholesterol synthesis
Fatty acid oxidation
mRNA metabolism
Peroxisomal lipid metabolism
Protein metabolism
Purine metabolism
Pyrimidine metabolism
Ribonucleotide synthesis
RNA metabolism
Sphingolipid metabolism
Steroid metabolism
Steroid synthesis
TCA cycle
UCS
MUC17
PTEN
CHD4
FBXW7
AHNAK
ARID1A
PPP2R1A
Gluconeogenesis
Glycogenolysis
Peroxisomal lipid metabolism
Ribonucleotide synthesis
Sphingolipid metabolism
TCA cycle
Fatty acid oxidation
Carbohydrate Metabolism
UVM
SF3B1
EIF1AX
BAP1
GNA11
GNAQ
mRNA metabolism
RNA metabolism
Glycolysis
NO metabolism
Biological Oxidation

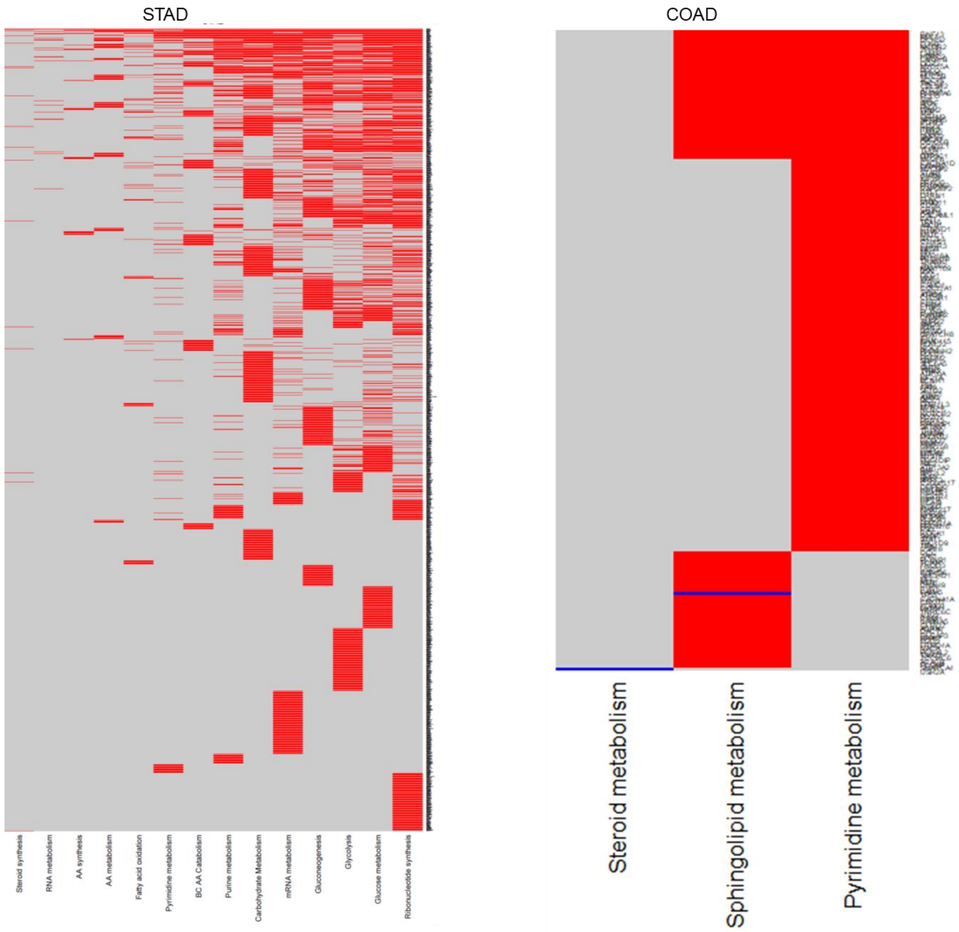

Supplementary Figure 6. Metabolism-related genes for each cancer type. All metabolism-related genes (y-axis) are presented with metabolic signatures (x-axis) for each cancer type. Red squares represent genes significantly more mutations in tumors with high metabolic signatures. Blue squares represent genes significantly fewer mutations in tumor with high metabolic signature.

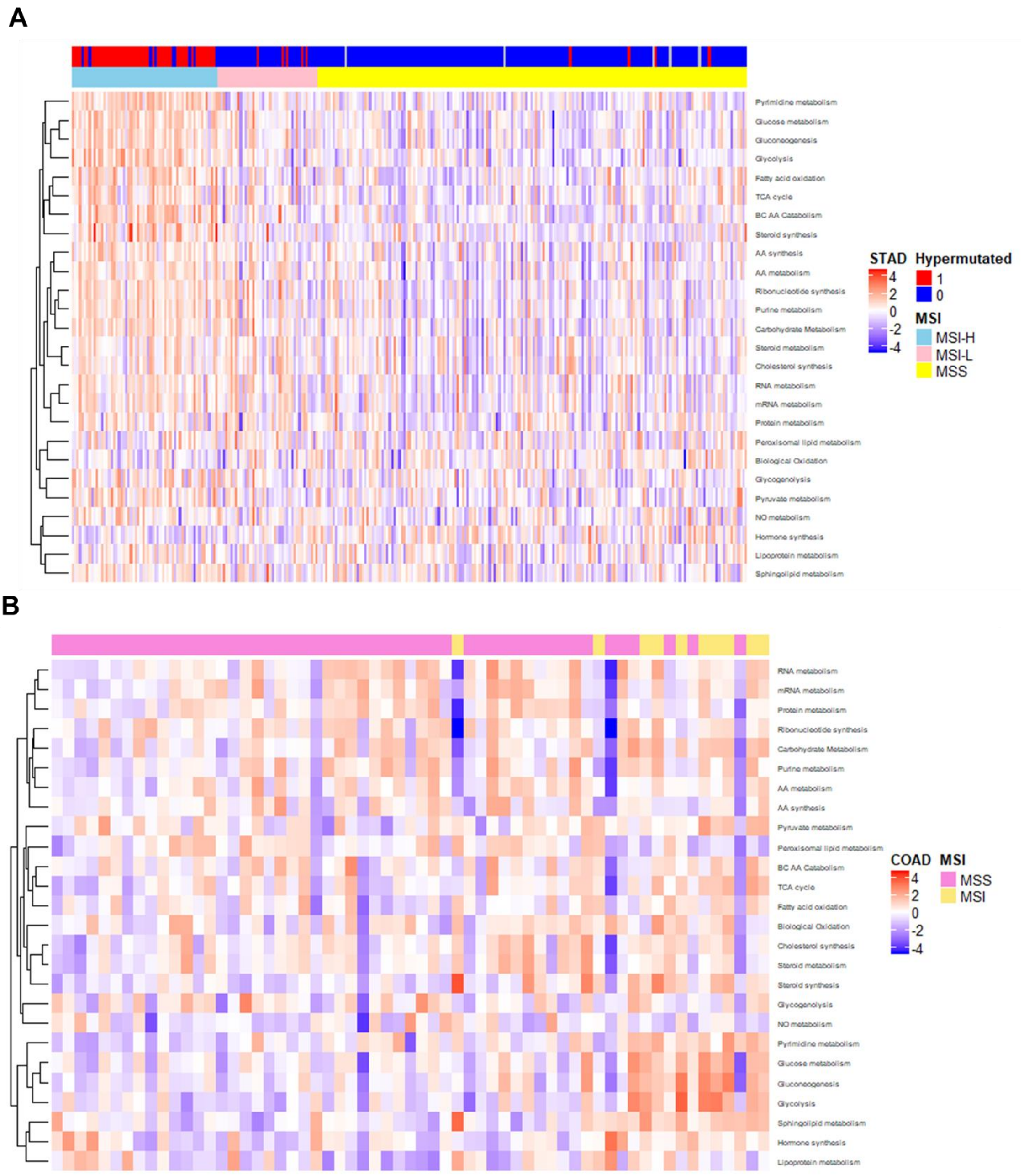

Supplementary Figure 7. Difference in metabolic signatures according to the presence of microsatellite instability. The heatmap depicting metabolic signatures of stomach adenocarcinoma (STAD) (A) and colon adenocarcinoma (COAD) (B). The presence of microsatellite instability or hypermutation are shown above the heatmap (MSS =

microsatellite stable; MSI = microsatellite instable; MSI-H = High microsatellite instability; MSI-L = Low microsatellite instability).

Supplementary Tables

Supplementary Table 1. List of metabolic pathways from Reactome used in the study

**Metabolic pathways from Reactome**	**Abbreviation**
Metabolism of amino acids	AA metabolism
Amino acid synthesis and interconversion	AA synthesis
Branched chain amino acid catabolism	BC AA Catabolism
Biological oxidations	Biological Oxidation
Metabolism of carbohydrates	Carbohydrate Metabolism
Cholesterol biosynthesis	Cholesterol synthesis
Mitochondrial fatty acid beta oxidation	Fatty acid oxidation
Glyconeogenesis	Gluconeogenesis
Glucose metabolism	Glucose metabolism
Glycogen breakdown glycogenolysis	Glycogenolysis
Glycolysis	Glycolysis
Hormone synthesis	Hormone synthesis
Lipoprotein metabolism	Lipoprotein metabolism
Metabolism of mRNA	mRNA metabolism
Metabolism of nitric oxide	NO metabolism
Peroxisomal lipid metabolism	Peroxisomal lipid metabolism
Metabolism of proteins	Protein metabolism
Purine metabolism	Purine metabolism
Pyrimidine metabolism	Pyrimidine metabolism
Pyruvate metabolism	Pyruvate metabolism
Purine ribonucleotide monophosphate biosynthesis	Ribonucleotide synthesis
Metabolism of RNA	RNA metabolism
Sphingolipid metabolism	Sphingolipid metabolism
Steroid metabolism	Steroid metabolism
Steroid hormone biosynthesis	Steroid synthesis
Citric acid cycle	TCA cycle

Supplementary Table 2. Number of samples for each cancer subtype included for the analysis

Disease type	Abbreviation	Number of samples
Adenocortical carcinoma	ACC	54
Bladder Urothelial Carcinoma	BLCA	390
Breast invasive carcinoma	BRCA	971
Cervical Squamous Cell Carcinoma and Endocervical Adenocarcinoma	CESC	193
Cholangiocarcinoma	CHOL	35
Colon Adenocarcinoma	COAD	208
Diffuse large B-cell lymphoma	DLBC	48
Esophageal Carcinoma	ESCA	184
Glioblastoma multiforme	GBM	142
Head and Neck Squamous Cell Carcinoma	HNSC	503
Kidney Chromophobe	KICH	66
Kidney Renal Clear Cell Carcinoma	KIRC	433
Kidney Renal Papillary Cell Carcinoma	KIRP	281
Brain lower grade glioma	LGG	516
Liver Hepatocellular Carcinoma	LIHC	367
Lung Adenocarcinoma	LUAD	478
Lung squamous cell carcinoma	LUSC	176
Ovarian serous cystadenocarcinoma	OV	248
Pancreatic Adenocarcinoma	PAAD	119
Pheochromocytoma and Paraganglioma	PCPG	179
Prostate Adenocarcinoma	PRAD	497
Rectum adenocarcinoma	READ	53
Sarcoma	SARC	245
Stomach Adenocarcinoma	STAD	370
Testicular germ cell tumors	TGCT	147
Thyroid carcinoma	THCA	493
Thymoma	THYM	115
Uterine Carcinosarcoma	UCS	57
Uveal melanoma	UVM	80

SUPPLEMENTARY REFERENCES

1. Colaprico A, Silva TC, Olsen C, Garofano L, Cava C, Garolini D, Sabedot TS, Malta TM, Pagnotta SM, Castiglioni I: **TCGAbiolinks: an R/Bioconductor package for integrative analysis of TCGA data.** *Nucleic acids research* 2015, **44:**e71-e71.
2. Mayakonda A, Koeffler HP: **Maftools: Efficient analysis, visualization and summarization of MAF files from large-scale cohort based cancer studies.** *BioRxiv* 2016**:**052662.
3. Croft D, Mundo AF, Haw R, Milacic M, Weiser J, Wu G, Caudy M, Garapati P, Gillespie M, Kamdar MR: **The Reactome pathway knowledgebase.** *Nucleic acids research* 2013, **42:**D472-D477.
4. Barbie DA, Tamayo P, Boehm JS, Kim SY, Moody SE, Dunn IF, Schinzel AC, Sandy P, Meylan E, Scholl C: **Systematic RNA interference reveals that oncogenic KRAS-driven cancers require TBK1.** *Nature* 2009, **462:**108.
5. Liberzon A, Subramanian A, Pinchback R, Thorvaldsdóttir H, Tamayo P, Mesirov JP: **Molecular signatures database (MSigDB) 3.0.** *Bioinformatics* 2011, **27:**1739-1740.
6. Hänzelmann S, Castelo R, Guinney J: **GSVA: gene set variation analysis for microarray and RNA-seq data.** *BMC bioinformatics* 2013, **14:**7.
7. Maaten Lvd, Hinton G: **Visualizing data using t-SNE.** *Journal of machine learning research* 2008, **9:**2579-2605.
8. Chung I-F, Chen C-Y, Su S-C, Li C-Y, Wu K-J, Wang H-W, Cheng W-C: **DriverDBv2: a database for human cancer driver gene research.** *Nucleic acids research* 2015, **44:**D975-D979.
